# Research on Covert Communication in Satellite–Ground-Integrated Sensor Networks Based on FH-DL-MPWFRFT

**DOI:** 10.3390/s26123716

**Published:** 2026-06-11

**Authors:** Lei Ni, Yichao Cai, Xiaobai Li, Hang Hu, Zheng Chu, Yuzhi Qi

**Affiliations:** 1 Early Warning Intelligence Department, Air Force Early Warning Academy, Wuhan 430019, China; yichaocai@163.com (Y.C.); lxbcici@163.com (X.L.); qiyuzhixd@163.com (Y.Q.); 2 College of Information and Navigation, Air Force Engineering University, Xi’an 710077, China; xd_huhang@126.com; 3 Next-Generation Internet of Everything Laboratory, Department of Electrical and Electronic Engineering, University of Nottingham Ningbo China, Ningbo 315100, China; andrew.chuzheng7@gmail.com

**Keywords:** satellite–ground-integrated sensor network, weighted fractional Fourier transform, polarization modulation, physical layer security

## Abstract

To further enhance the covert communication capability of satellite–ground-integrated sensor networks, a dual-polarization constellation joint modulation scheme based on frequency-hopping double-layer multi-parameter weighted fractional Fourier transform (FH-DL-MPWFRFT) is proposed from the perspective of physical layer security. The proposed scheme integrates the constellation confusion property of weighted fractional Fourier transform (WFRFT) with the anti-interception capability of frequency-hopping (FH) phase scrambling. Specifically, the weighted parameters of conventional 4-WFRFT are extended to construct a multi-parameter and multi-layer signal representation, and FH phase scrambling is introduced to realize dynamic constellation rotation and phase-domain encryption. Furthermore, a secure transmission model for satellite–ground-integrated sensor networks is established, revealing the constellation optimization principle and the fission-fusion mechanism of dual-polarization signals. Simulation results show that, compared with the non-FH benchmark, the proposed scheme significantly improves waveform-level anti-interception performance; even when eavesdropper obtains the modulation scheme and partial transform parameters, the symbol error rate (SER) of quadrature phase shift keying (QPSK) and four-phase modulation (4PM) signals remains around 0.4 to 0.5 under parameter mismatch, indicating that effective demodulation is difficult to achieve.

## 1. Introduction

With the advancement of sixth-generation (6G) technologies, the development of integrated air-space-ground communication networks is accelerating rapidly. In parallel, the continuous evolution of space-air-ground integrated information networks has rendered satellite–ground-integrated sensor networks an indispensable infrastructure for intelligent sensing and ubiquitous connectivity [1,2,3,4]. By leveraging the wide coverage and high mobility of satellite communication systems, together with the flexible deployment, cost efficiency, and strong environmental sensing capability of terrestrial wireless sensor networks, such networks demonstrate significant application potential in military reconnaissance, disaster monitoring, ocean observation, emergency communications, and information provision for remote areas. However, due to the open wireless propagation environment and long-distance transmission nature of satellite–ground-integrated sensor networks, transmitted signals are vulnerable to illegal interception, malicious jamming, signal detection, and cryptanalysis [4,5]. These threats correspond to different but related security requirements: secure transmission focuses on the reliable and confidential delivery of information, anti-interception emphasizes the resistance of transmitted waveforms to detection, identification, parameter estimation, and unauthorized demodulation, whereas covert communication further aims to conceal the existence or distinguishable characteristics of the communication process itself. In complex electromagnetic environments and high-adversarial scenarios, conventional communication schemes fail to simultaneously satisfy these requirements, especially the stringent covertness requirement. Accordingly, improving the covert communication capability of satellite–ground-integrated sensor networks, supported by secure transmission and anti-interception mechanisms, has become an urgent research priority [6,7,8].

Currently, satellite communication systems and wireless sensor networks mainly rely on upper-layer cryptographic algorithms (such as symmetric encryption and public-key encryption schemes) as well as anti-jamming techniques including spread spectrum and frequency-hopping (FH) to ensure communication security [9]. However, these methods primarily operate at the protocol and key management levels, leading to high computational complexity, challenges in key distribution, and vulnerability to detection and identification in highly adversarial environments. Meanwhile, traditional modulation schemes possess fixed constellation structures and distinct signal features, which can be readily identified through feature extraction and parameter estimation techniques. Recently, Transformer- and large language model (LLM)-based security methods have been introduced into wireless communications for signal detection, modulation recognition, intrusion detection, and intelligent security management [10,11,12,13]. Although these data-driven approaches improve feature extraction and decision-making capability, their performance generally depends on training data, model generalization, computational resources, and prior knowledge of the electromagnetic environment. Therefore, they mainly enhance security from the perspectives of detection, recognition, and management, whereas waveform-domain protection at the signal generation stage still requires further investigation. To address these challenges, physical layer security has emerged as an important research direction in wireless communication security in recent years [14,15,16]. By optimizing waveform structures, modulation schemes, and channel characteristics, physical layer security has great potential to achieve “perfect secrecy” and is identified as a significant complement to traditional cryptographic and intelligent security techniques. Therefore, introducing physical layer security technologies into satellite–ground-integrated sensor networks is of great significance for improving overall system security performance.

The fractional Fourier transform (FRFT), as a generalized transform between the time and frequency domains, provides adjustable transform orders and multidimensional signal representation capability. Furthermore, the weighted fractional Fourier transform (WFRFT) was initially proposed by Shih in 1995, and Shih [14] pioneered the introduction of WFRFT theory into optical communication systems. The WFRFT constructs signals by forming weighted combinations of multiple FRFT components with distinct orders, which enables diversified signal representations across multiple transform domains and facilitates constellation reconstruction and scrambling. Consequently, WFRFT-based modulation possesses significant constellation variability and high parametric flexibility and has been applied in the field of covert communication. According to [15], by extending the weighting parameters of the conventional 4-WFRFT structure, signal processing diversity and modulation adaptability can be further enhanced [16]. Existing studies indicate that WFRFT-modulated satellite signals exhibit constellation rotational ambiguity, uniform bit-energy distribution, and strong resistance to parameter scanning, demonstrating advantages in anti-jamming and anti-interception performance [17,18]. In [19], a unified framework based on WFRFT and orthogonal time frequency space (OTFS) is proposed in multi-device cooperative integrated sensing and communication (ISAC) systems, and the multi-WFRFT (MWFRFT) theory is introduced into generalized spatial modulation (GSM) systems for the first time to enhance physical layer security in [20]. However, most current research focuses on single-polarization or single-layer structural designs and has not fully incorporated the practical characteristics of satellite–ground-integrated networks into a systematic security modeling framework.

On the other hand, chaotic mappings possess properties such as sensitivity to initial conditions, pseudo-randomness, and unpredictability, which make them promising for secure communications. By employing a chaotic map in the design of the FH sequence, trajectory encryption and phase scrambling can be realized, thus strengthening the signal’s resistance to cryptanalysis [21]. Nevertheless, a single chaotic encryption mechanism still exhibits limitations when confronted with advanced signal processing and parameter estimation algorithms and, thus, requires integration with transform-domain modulation techniques to further improve overall security performance. In addition, modern satellite communication systems employ dual-polarization technology to enhance spectral efficiency and system capacity. However, the potential of dual-polarization resources in secure modulation design has not yet been fully exploited. Leveraging dual-polarization characteristics to construct multi-layer signal structures and achieve constellation fission and fusion mechanisms constitutes another important research direction for enhancing physical layer security in satellite–ground-integrated sensor networks.

In summary, given the complex electromagnetic environments and severe adversarial threats encountered in satellite–ground-integrated sensor networks, it is imperative to develop a physical-layer secure communication scheme that combines the constellation confusion capability of the WFRFT with encryption mechanisms based on FH phase scrambling. By extending WFRFT weighting parameters, introducing multi-layer signal design, and adopting a dual-polarization joint modulation framework, this paper establishes a secure transmission model for satellite–ground-integrated sensor networks, reveals the underlying mechanisms of constellation optimization and fission-fusion processes, and validates the security performance through simulation experiments. The proposed approach provides theoretical foundations and technical support for enhancing covert communication performance in space-ground integrated networks by improving waveform-level anti-interception capability and physical-layer secure transmission performance.

The major contributions to this paper are listed as follows:

First, considering the security vulnerabilities of conventional communication schemes in satellite–ground-integrated sensor networks—including high computational complexity of upper-layer cryptography and weak anti-interception performance of traditional modulations—a physical layer security framework combining WFRFT and FH phase scrambling is proposed. This framework enables integrated constellation scrambling and signal encryption at the physical layer.

Second, by extending the weighting parameters of the traditional 4-WFRFT structure and introducing a dual-polarization joint modulation mechanism, a multi-layer and multi-dimensional secure signal model adapted to the practical characteristics of satellite–ground-integrated networks is constructed, which effectively enhances the diversity of signal representation and the difficulty of interception.

Third, the underlying mechanisms of constellation design, fission and fusion in the proposed secure transmission model, are systematically revealed, clarifying the interaction law between the double-layer MPWFRFT (DL-MPWFRFT) constellation confusion, FH phase scrambling, and dual-polarization modulation, and providing a theoretical basis for the design of satellite covert communication.

Fourth, simulation experiments are carried out to verify the security and reliability of the proposed scheme. The results demonstrate that the scheme has excellent anti-interception, anti-jamming, and anti-decoding performance, which can effectively improve the overall security level of satellite–ground-integrated sensor networks.

## 2. Preliminary

### 2.1. System Model

**Figure 1:**
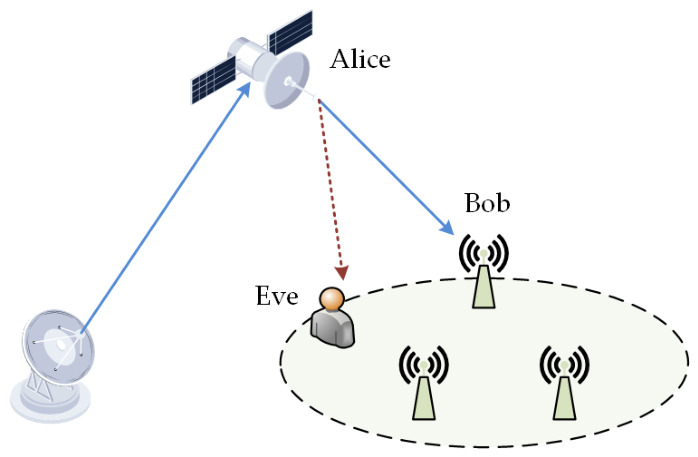
The system model.

We consider constructing a satellite–ground-integrated sensor network, as illustrated in Figure 1. The system model consists of one satellite transmitter (Alice), a wireless sensor network with *n* legitimate receivers (Bobs), and an eavesdropper (Eve). All of these nodes are equipped with dual-polarized antennas, which are capable of simultaneous transmission and reception of orthogonal dual-polarized signals. It is assumed that Eve is located within the coverage area of the satellite communication and possesses identical signal receiving and processing capabilities as Bob. If the satellite transmits signals without adopting any security measures, Eve can also demodulate the information, resulting in a confidential information leakage.

Polarization modulation (PM) encodes information via the amplitude ratio and phase difference in the polarization domain of signals. It can be combined with conventional amplitude-frequency domain modulation schemes to design multi-dimensional joint modulation schemes with higher transmission efficiency, thereby significantly improving the communication capacity and transmission energy efficiency of the system. By employing a wireless transmission scheme based on the polarization-amplitude-phase modulation (PAPM) model [22], the transmitted signal $x_{k}$ at Alice during the *k*-th symbol interval at the transmitter can be expressed as follows:

(1) $$x_{k}=\left[\begin{array}{c} x_{kH}\\ x_{kV} \end{array}\right]=\left[\begin{array}{c} \cos\gamma_{k}\\ \sin\gamma_{k}\;e^{j\phi_{k}} \end{array}\right]A_{k}e^{j(\omega_{c}t+\theta_{k})},$$

where k=1,2,3…N, $x_{kH}$ and $x_{kV}$ denote the horizontal and vertical components of the transmitted signal $x_{k}$, respectively. $\gamma_{k}$ and $\phi_{k}$ represent the polarization phase descriptors, with $\gamma_{k}\in \left[0,\pi /2\right]$ and $\phi_{k}\in \left[0,2\pi \right]$. $A_{k}$ and $\theta_{k}$ correspond to the amplitude and phase of the amplitude-modulated signal, respectively, and $\omega_{c}$ denotes the carrier frequency.

In our study, we focus on the secure transmission with satellite signal constellation splitting, and the channel state information (CSI) is not emphasized. In practice, the effect of polarization-dependent loss (PDL) can be eliminated by the pre-compensation method and zero-forcing prefiltering matrix method [23]. To facilitate the analysis, the satellite channel is assumed to be an ideal additive white Gaussian noise (AWGN) channel. Then, the received signal $y_{k}$ at the receiver during the *k*-th symbol interval is given by:

(2) $$\begin{array}{cc} y_{k} & =\left[\begin{array}{c} y_{kH}\\ y_{kV} \end{array}\right]=\sqrt{\rho_{k}}x_{k}+n_{k}\\ & =\sqrt{\rho_{k}}\left[\begin{array}{c} x_{kH}\\ x_{kV} \end{array}\right]+\left[\begin{array}{c} n_{kH}\\ n_{kV} \end{array}\right],\;k=1,2,3,\ldots ,N, \end{array}$$

where $\rho_{k}$ denotes the transmit power, and $n_{k}$ represents the AWGN vector.

After receiving the signal $y_{k}$, Bob can obtain the received polarization phase descriptors ${\overset{⌢}{\gamma }}_{kR}$ and ${\overset{⌢}{\phi }}_{kR}$ according to (3). Then, by employing the maximum likelihood criterion to compare with the PM constellation at the transmitter, the final polarization phase descriptors $\gamma_{kR}^{\prime }$ and $\phi_{kR}^{\prime }$ are obtained, from which the polarization-domain information is retrieved.

(3) $${\overset{⌢}{\gamma }}_{kR}=\arctan\frac{\left|y_{kV}\right|}{\left|y_{kH}\right|},$$

(4) $${\overset{⌢}{\phi }}_{kR}=\vartheta (y_{kV})-\vartheta (y_{kH}),$$

where |·| denotes the modulus operation, and ϑ(·) represents the phase extraction operation.

Furthermore, the amplitude-phase modulated signal $y_{k}^{\mathrm{APM}}$ is derived via (5), and the time-domain information is subsequently obtained according to the time-domain modulation criterion.

(5) $$y_{k}^{\mathrm{APM}}={\left[\begin{array}{c} \cos\gamma_{kR}^{\prime }\\ \sin\gamma_{kR}^{\prime }\;e^{j\phi_{kR}^{\prime }} \end{array}\right]}^{H}y_{k},$$

where ${\left(\cdot \right)}^{H}$ stands for the Hermitian transpose operations.

### 2.2. Mathematical Foundation of DL-MPWFRFT

For an arbitrary complex sequence $X_{0}$, the α-th order 4-WFRFT is defined as [24]

(6) $$\begin{array}{cc} Y & =F^{\alpha ,V}\;\left[X_{0}\right]=\omega_{0}\left(\alpha ,V\right)X_{0}+\omega_{1}\left(\alpha ,V\right)X_{1}\\ \; & \;+\omega_{2}\left(\alpha ,V\right)X_{2}+\omega_{3}\left(\alpha ,V\right)X_{3}, \end{array}$$

where $F^{\alpha ,V}$ denotes the WFRFT operator, $\alpha \in \left[-2,2\right]$ is the transform order, and $X_{l}\;\left(l=0,1,2,3\right)$ are the 0-th to 3rd discrete Fourier transform (DFT) of $X_{0}$, respectively. The corresponding weighting coefficients $\omega_{l}$ are given by

(7) $$\left\{\begin{array}{l} \omega_{l}\left(\alpha ,V\right)=\frac{1}{4}\sum_{k=0}^{3}\exp\;\left\{\pm \frac{2\pi i}{4}\left[\left(4m_{k}+1\right)\alpha \left(k+4n_{k}\right)-lk\right]\right\}\\ V=\left[\mathrm{MV},\;\mathrm{NV}\right] \end{array}\right.$$

where $\mathrm{MV}=\left[m_{0},m_{1},m_{2},m_{3}\right]$, $\mathrm{NV}=\left[n_{0},n_{1},n_{2},n_{3}\right]$ are scaling vectors, and all values are integers. For the plus-minus sign ± in (7), the weighting coefficients $\omega_{l}$ rotate with the variation of parameter α on the complex plane, where the positive sign denotes counterclockwise rotation, and the negative sign denotes clockwise rotation.

It should be noted that, when V=0, only one parameter α is involved in (6), which is referred to as the single-parameter WFRFT (SPWFRFT). Moreover, when V≠0, multiple independent parameters are adopted the weighting coefficients in the equation are extended from one parameter to multiple parameters, yielding the multiple-parameters WFRFT (MPWFRFT). Studies have shown that the SPWFRFT signal exhibits constellation rotation and splitting characteristics [25], whose transformation behavior depends on the selection of the parameter α. As a generalized form of the 4-WFRFT, the MPWFRFT also features constellation rotation and constellation splitting. The anti-interception performance hinges on the degree of constellation diffusion and confusion introduced by the time-frequency transform. The increased number of transform parameters enriches the variation patterns of the MPWFRFT signal constellation, making it applicable to covert communication [26].

**Figure 2:**
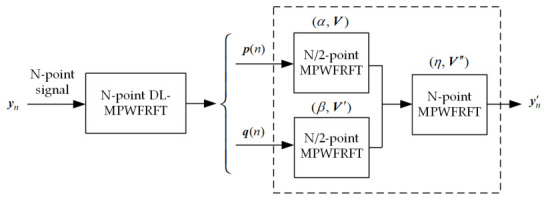
Schematic diagram of DL-MPWFRFT.

On the basis of MPWFRFT, the transmitted signal $y_{n}$ is divided into two parts, $p_{n}$ and $q_{n}$, where the lengths of these two signals can be set as required. In our work, we assume that the signal lengths of both $p_{n}$ and $q_{n}$ are N/2. Moreover, MPWFRFT is performed on each part separately. Finally, the transformed signals are concatenated, and another MPWFRFT transform is applied to obtain the DL-MPWFRFT signal $y_{n}^{\prime }$. The detailed principle of the transformation is illustrated in Figure 2.

For the DFT matrix $F_{N}$, its expression can be written as:

(8) $$F_{N}=\frac{1}{\sqrt{N}}{\left[\begin{array}{cccc} 1 & 1 & \ldots & 1\\ 1 & e^{\frac{-j2\pi }{N}} & \ldots & e^{\frac{-j2\pi (N-1)}{N}}\\ \vdots & \vdots & e^{\frac{-j2\pi (n-1)(k-1)}{N}} & \vdots \\ 1 & e^{\frac{-j2\pi (N-1)}{N}} & \ldots & e^{\frac{-j2\pi {\left(N-1\right)}^{2}}{N}} \end{array}\right]}_{N\times N},$$

where the element located at the *n*-th row and *k*-th column of matrix $F_{N}$ is expressed as $e^{\frac{-j2\pi (n-1)(k-1)}{N}}$, *n* and *k* take values of 1,2,3,…,N.

The normalized Fourier matrix is unitary with the following properties:

(9) $${\left(F_{N}\right)}^{0}=I_{N},{\left(F_{N}\right)}^{2}=T_{N},{\left(F_{N}\right)}^{3}=F_{N}^{-1},$$

where $I_{N}$ denotes an N×N identity matrix, $T_{N}$ denotes the shift matrix, and its expression is:

(10) $$T_{N}=F_{N}^{2}={\left[\begin{array}{cccccc} 1 & 0 & \cdots & 0 & 0 & 0\\ 0 & 0 & \cdots & 0 & 0 & 1\\ 0 & 0 & \cdots & 0 & 1 & 0\\ \vdots & \cdots & 0 & \ddots & 0 & 0\\ 0 & 0 & 1 & 0 & \cdots & 0\\ 0 & 1 & 0 & 0 & \cdots & 0 \end{array}\right]}_{N\times N}$$

Then, the corresponding N/2-point MPWFRFT can be expressed by:

(11) $$\begin{array}{cc} \; & F_{\alpha }^{V}\left[p\left(n\right)\right]=[\omega_{0}\left(\alpha ,V\right)I_{N}+\omega_{1}\left(\alpha ,V\right)F_{N}+\omega_{2}\left(\alpha ,V\right)T_{N}\\ \; & \;+\omega_{3}\left(\alpha ,V\right)F_{N}^{-1}]p\left(n\right)=\xi_{N/2}\left(\alpha ,V\right)p\left(n\right), \end{array}$$

(12) $$\begin{array}{cc} \; & F_{\beta }^{V^{\prime }}\left[q\left(n\right)\right]=[\omega_{0}\left(\beta ,V^{\prime }\right)I_{N}+\omega_{1}\left(\beta ,V^{\prime }\right)F_{N}+\omega_{2}\left(\beta ,V^{\prime }\right)T_{N}\\ \; & \;+\omega_{3}\left(\beta ,V^{\prime }\right)F_{N}^{-1}]q\left(n\right)=\xi_{N/2}\left(\beta ,V^{\prime }\right)q\left(n\right), \end{array}$$

where α and V, β and $V^{\prime }$ represent the transform order and scale vector of their respective MPWFRFT, respectively. $\xi_{N/2}\left(\alpha ,V\right)$ and $\xi_{N/2}\left(\beta ,V^{\prime }\right)$ indicate the corresponding MPWFRFT transformation matrix.

Let $m\left(n\right)={\left[\xi_{N/2}\left(\alpha ,V\right)p\left(n\right),\xi_{N/2}\left(\beta ,V^{\prime }\right)q\left(n\right)\right]}^{T}$, since both $\xi_{N/2}\left(\alpha ,V\right)p\left(n\right)$ and $\xi_{N/2}\left(\beta ,V^{\prime }\right)q\left(n\right)$ have a length of N/2 points, the concatenated sequence m(n) has a length of *N* points , and its corresponding *N*-point MPWFRFT expression is shown as follows:

(13) $$\begin{array}{cc} F_{\eta }^{V^{\prime \prime }}\left[m\left(n\right)\right]\; & =[\omega_{0}\left(\eta ,V^{\prime \prime }\right)I_{N}+\omega_{1}\left(\eta ,V^{\prime \prime }\right)F_{N}+\omega_{2}\left(\eta ,V^{\prime \prime }\right)T_{N}\\ \; & \;+\omega_{3}\left(\eta ,V^{\prime \prime }\right)F_{N}^{-1}]m\left(n\right)\\ \; & =\xi_{N}\left(\eta ,V^{\prime \prime }\right)m\left(n\right)\\ \; & =\xi_{N}\left(\eta ,V^{\prime \prime }\right)\left[\begin{array}{c} \xi_{N/2}\left(\alpha ,V\right)p\left(n\right)\\ \xi_{N/2}\left(\beta ,V^{\prime }\right)q\left(n\right) \end{array}\right]\\ \; & =\xi_{N}\left(\eta ,V^{\prime \prime }\right)\left[\begin{array}{cc} \xi_{N/2}\left(\alpha ,V\right) & 0\\ 0 & \xi_{N/2}\left(\beta ,V^{\prime }\right) \end{array}\right]\left[\begin{array}{c} p(n)\\ q(n) \end{array}\right]\\ \; & =\xi_{DL}\left(\alpha ,V,\beta ,V^{\prime },\eta ,V^{\prime \prime }\right)y\left(n\right), \end{array}$$

where η and $V^{\prime \prime }$ represent the transform order and scale vector of the second-layer MPWFRFT, and $\xi_{DL}\left(\alpha ,V,\beta ,V^{\prime },\eta ,V^{\prime \prime }\right)$ denotes the DL-MPWFRFT transform matrix.

By definition, the DL-MPWFRFT extends the transform layers and increases the number of transform parameters from 9 in MPWFRFT to 27, yielding richer transformation diversity and stronger signal transmission security. Benefiting from its Gaussian-like distribution, the covert signal can be superimposed on existing satellite service channels to realize overlapped communication. Structurally, DL-MPWFRFT comprises three MPWFRFT modules and only introduces an extra scaling vector at the parameter input compared with the standard WFRFT. As MPWFRFT unifies single-carrier and multi-carrier systems, DL-MPWFRFT signals can also be interpreted as a hybrid of both components. Moreover, its physical implementation schematic indicates low complexity, making it favorable for practical deployment.

**Figure 3:**
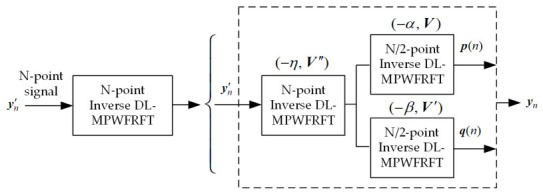
Schematic diagram of the inverse DL-MPWFRFT.

The schematic diagram of the inverse DL-MPWFRFT is illustrated in Figure 3, which intuitively presents the complete implementation process and key operation steps of the inverse transform. Analogous to the MPWFRFT, the inverse DL-MPWFRFT can be implemented simply by negating the transform orders at corresponding positions while retaining the scaling vectors unchanged, followed by the corresponding MPWFRFT operations to recover the original transmitted signal y(n).

## 3. FH-DL-MPWFRFT-Based Dual-Polarization Satellite Secure Transmission System

### 3.1. Frequency-Hopping Phase Scrambling

FH technology is a core technique for secure satellite transmission and anti-jamming communication [27]. Motivated by the FH mechanism, the key implementation of FH phase scrambling relies on the interaction between a random FH sequence and the phase of the transmitted signal. Its principle can be summarized as follows: first, a random FH sequence is generated (e.g., via chaotic mapping or pseudo-random sequence generators, ensuring unpredictability and randomness). This sequence serves as the control signal for phase scrambling and acts on the phase dimension of the signal to be transmitted. Since the random FH sequence is a discrete numerical sequence that cannot directly modulate the signal phase; it must be mapped into a phase factor via a dedicated conversion scheme for phase modulation.

**Figure 4:**
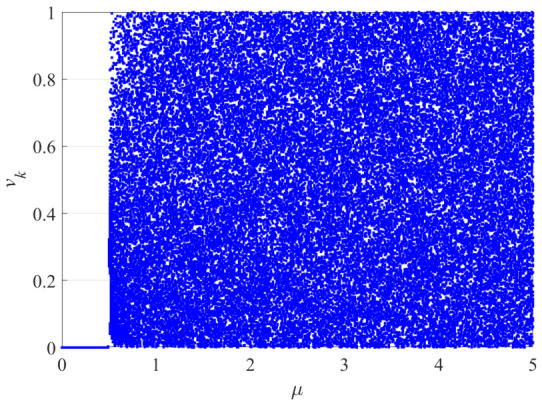
Bifurcation diagram of the proposed chaotic map.

Let the generated random FH sequence be ${\left\{v_{k}\right\}}_{k=1}^{N}$, where *k* is the sequence index k=1,2,…,N, *N* is the length of the FH sequence, and $v_{k}$ is the FH frequency value at the *k*-th moment. In the conventional FH technique, the carrier frequency hops periodically according to a pseudo-random (PN) code. Linear codes such as *m*-sequence and Gold sequence are used, which have the disadvantage of being vulnerable to cracking by linear prediction [28]. In our work, to improve the anti-decryption performance against eavesdropping, the FH sequence is constructed based on a chaotic map. Different from the one-dimensional logistic map used in [29], a new design method of fifth-order chaotic mapping is proposed, as detailed below:

(14) $$v_{k+1}=\mathrm{mod}\left(\mu v_{k}^{5}+2\mu v_{k},\;1\right),$$

where $v_{0}$ is the initial value, μ denotes the fractal coefficient, and the mod operation only retains the decimal part between 0 and 1, i.e., forcibly constrains the result to the interval $\left[0,1\right)$. Here, the initial value is set to $v_{0}=0.9$, and the bifurcation diagram of the proposed chaotic map is obtained as Figure 4.

**Figure 5:**
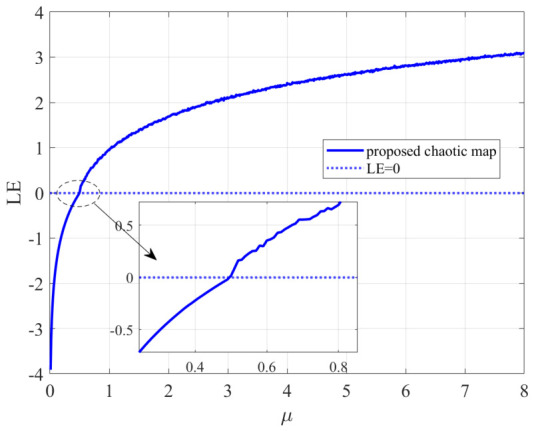
LE characteristic diagram of the proposed chaotic map.

The bifurcation diagram of the chaotic map reflects its dynamic properties and chaotic evolution. Ergodicity, a key characteristic of chaotic maps, means chaotic sequences are uniformly distributed within their value range without obvious aggregation or gaps, ensuring their randomness and unpredictability. Better ergodicity makes the sequence’s statistical characteristics closer to an ideal random sequence, enhancing its resistance to attackers’ statistical analysis. It can be observed from Figure 4 that for the proposed chaotic map, when the fractal coefficient μ>0.5, the chaotic map enters a chaotic state and exhibits a full mapping state. To further explore the properties of the new chaotic map, we analyze its Lyapunov exponent (LE) characteristics. The LE value is one of the characteristic quantities describing the behavior of dynamical systems and can also measure the system’s sensitivity to initial values. For a chaotic system, the LE value must be greater than 0, and its mathematical expression is given by [30]:

(15) $$\mathrm{LE}=\lim_{n\to \infty }\frac{1}{n}\sum_{k=0}^{n-1}\ln\left|\frac{df^{n}\left(v_{k}\right)}{dv}\right|=\lim_{n\to \infty }\frac{1}{n}\sum_{k=0}^{n-1}\ln\left|f^{\prime }(v_{k})\right|,$$

where $v_{k}=f\left(v_{k-1}\right)$, and the LE characteristics of the proposed chaotic map are shown in Figure 5. It can be seen that, when the fractal coefficient μ>0.5, all the corresponding Lyapunov exponents are greater than 0. This indicates that the proposed new chaotic map meets the requirements of chaotic systems and exhibits superior performance.

When the generated FH sequence acts on the phase of the transmitted signal, it will dynamically and randomly perturb and modulate the phase of the signal, which essentially completes the encryption processing of the transmitted signal. The FH phase scrambling factor can be obtained by the following formula:

(16) $$\varphi_{k}=\exp\left(j2\pi v_{k}\right),$$

**Figure 6:**
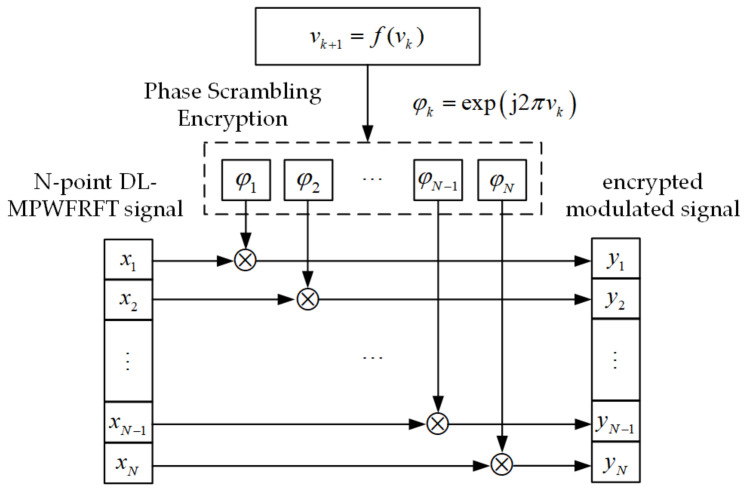
FH phase scrambling encryption diagram.

After phase scrambling, the phase characteristics of the signal are randomized, which deviates from the phase law of the original signal and cannot be directly restored to the original information. The specific implementation process is illustrated in Figure 6.

It can be seen that the specific FH sequence is obtained with the given initial value $v_{0}$. When applied to the phase of the transmitted signal, the sequence encrypts the signal. At this point, for an eavesdropper to recover the transmitted signal, it must first eliminate the effect of the proposed FH phase scrambling and decipher the chaotic map. However, due to the extreme sensitivity of chaotic maps to initial values, even a tiny error will result in an extremely high bit error rate. This increases the difficulty for Eve to crack the signal, thereby ensuring information security.

After processing with FH-DL-MPWFRFT, the received signal at the receiver is:

(17) $$\begin{array}{cc} y_{kR} & =\left[\begin{array}{c} y_{kRH}\\ y_{kRV} \end{array}\right]\\ \; & =\sqrt{\rho_{k}}\left[\begin{array}{c} \xi_{DL}\left(\Theta \right)\exp\;\left(j2\pi v_{k}\right)x_{kH}\\ \xi_{DL}\left(\Theta \right)x_{kV} \end{array}\right]+\left[\begin{array}{c} n_{kH}\\ n_{kV} \end{array}\right], \end{array}$$

where $\Theta ≜\left\{\alpha ,V,\beta ,V^{\prime },\eta ,V^{\prime \prime }\right\}$.

For the eavesdropper, assuming that the specific modulation scheme of the legitimate user is known, the signal after demodulation by Eve is:

(18) $$\begin{array}{cc} {\tilde{y}}_{kR} & =\sqrt{\rho_{k}}\left[\begin{array}{c} \xi_{DL}\left(\tilde{\Theta }\right)\xi_{DL}\left(\Theta \right)\exp\;\left(j2\pi (v_{k}-{\tilde{v}}_{k})\right)x_{kH}\\ \xi_{DL}\left(\tilde{\Theta }\right)\xi_{DL}\left(\Theta \right)x_{kV} \end{array}\right]\\ \; & \;+\left[\begin{array}{c} \xi_{DL}\left(\tilde{\Theta }\right)\exp\;\left(-j2\pi {\tilde{v}}_{k}\right)n_{kH}\\ \xi_{DL}\left(\tilde{\Theta }\right)n_{kV} \end{array}\right], \end{array}$$

where $\tilde{\Theta }≜\left\{\tilde{\alpha },\tilde{V},\tilde{\beta },{\tilde{V}}^{\prime },\tilde{\eta },{\tilde{V}}^{\prime \prime }\right\}$, which denotes Eve’s demodulation parameter set.

### 3.2. System Architecture

**Figure 7:**
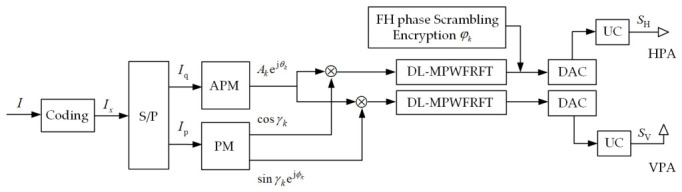
Schematic diagram of transmitter architecture.

Figure 7 shows the schematic diagram of the transmitter structure at Alice. It can be seen that after source coding and channel coding, the information *I* is divided into two parts $I_{q}$ and $I_{p}$ by serial-to-parallel (S/P) conversion. Subsequently, $I_{q}$ is split into a horizontal component signal and a vertical component signal through amplitude-phase modulation (APM), while $I_{p}$ obtains two polarization states via PM. Then, polarization state weighting is performed on the two signals to obtain the PAPM signal. On the basis of PAPM, DL-MPWFRFT modulation is simultaneously applied to the two orthogonal signals. In particular, the horizontal component of the signal is further encrypted by frequency hopping phase scrambling after that to change the original carried information. Finally, after digital-to-analog conversion (DAC) and up-conversion (UC), the two signals are transmitted through a Horizontal polarization antenna (HPA) and a vertical polarization antenna (VPA), respectively.

**Figure 8:**
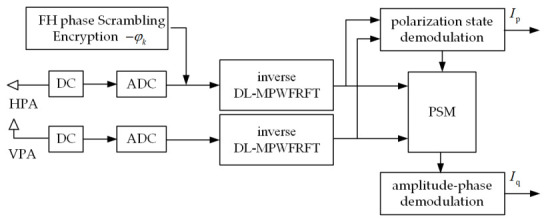
Schematic diagram of receiver architecture.

Figure 8 shows the schematic diagram of the receiver structure at Bob; the received signal is first processed by down-conversion (DC) and analog-to-digital conversion (ADC), followed by FH phase scrambling demodulation separately for the horizontally polarized signal, and DL-MPWFRFT demodulation, i.e., inverse DL-MPWFRFT, is performed on the two orthogonal signals simultaneously. Subsequently, polarization domain information is obtained through polarization state demodulation, and the APM signal is derived using the polarization state matching (PSM). Finally, amplitude-phase demodulation of the APM signal is carried out to obtain the time-domain information.

From a component-wise perspective, the PAPM and dual-polarization structure provide polarization-domain hiding by increasing the signal representation dimension and enabling constellation fission-fusion. The DL-MPWFRFT module introduces transform-domain constellation confusion through multi-layer weighted operations, thereby expanding the parameter space for unauthorized demodulation. The FH phase scrambling module further provides dynamic phase-domain protection by making the effective constellation time-varying. Therefore, the proposed scheme jointly achieves polarization-domain hiding, transform-domain confusion, and phase-domain scrambling, which enhances waveform-level anti-interception capability.

### 3.3. Computational Complexity Analysis

The computational complexity of the FH-DL-MPWFRFT method mainly originates from the DL-MPWFRFT operation because the multiplication of scrambling phases only increases linear complexity and does not change the overall complexity order of the DL-MPWFRFT method. For a sequence of length *N*, DL-MPWFRFT first splits it into two subsequences of length N/2, performs one N/2-point MPWFRFT on each subsequence, respectively, and finally conducts one *N*-point MPWFRFT after splicing. Accordingly, the total computational complexity is expressed as

(19) $$C_{DL}\left(N\right)=2C_{MP}\left(N/2\right)+C_{MP}\left(N\right)+O\left(N\right),$$

where the term O(N) accounts for the operations of sequence splitting, splicing, and weighted synthesis.

When implemented via fast Fourier transform (FFT), the *L*-point MPWFRFT only requires one FFT, one inverse FFT (IFFT), one cyclic shift, and several linear weighting operations. When radix-2 FFT is adopted, its computational complexity satisfies:

(20) $$C_{MP}\left(L\right)=2C_{FFT}\left(L\right)+O\left(L\right)=O\left(L\log L\right),$$

Substitute the above formula to derive:

(21) $$\begin{array}{cc} C_{\mathrm{DL}}\left(N\right) & =2\left[2C_{FFT}\left(N/2\right)+O\left(N/2\right)\right]+\left[2C_{FFT}\left(N\right)+O\left(N\right)\right]\\ \; & =4C_{FFT}\left(N/2\right)+2C_{FFT}\left(N\right)+O\left(N\right), \end{array}$$

Let $C_{FFT}\left(L\right)=cL\log_{2}L$, and *c* denotes a constant coefficient related to the specific FFT implementation, we can obtain:

(22) $$C_{DL}\left(N\right)=4cN\log_{2}N-2cN+O\left(N\right),$$

Therefore, the asymptotic computational complexity of DL-MPWFRFT implemented by FFT is $O(N\log_{2}N)$, which is of the same order of magnitude as that of the single-layer *N*-point WFRFT. The reason is that the superposition of two N/2-point transforms in the first layer and one *N*-point transform in the second layer still takes $N\log_{2}N$ as the dominant term. Therefore, richer constellation scrambling effects and more adjustable parameters are obtained without significantly increasing the computational overhead.

## 4. Simulation and Performance Analysis

### 4.1. Constellation Characteristics of DL-MPWFRFT

**Figure 9:**
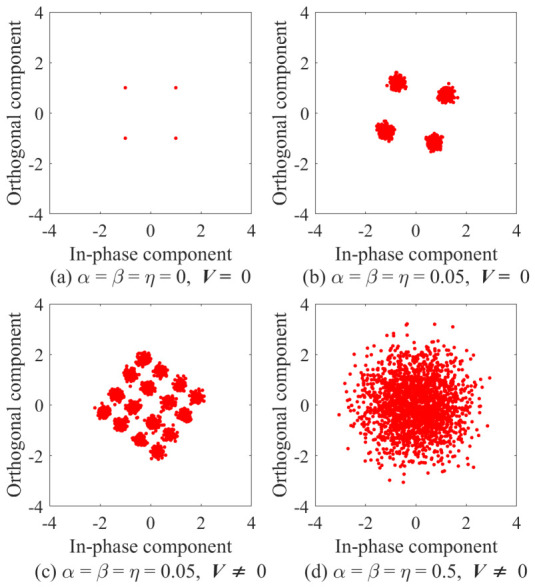
Characteristics of the QPSK constellation.

**Figure 10:**
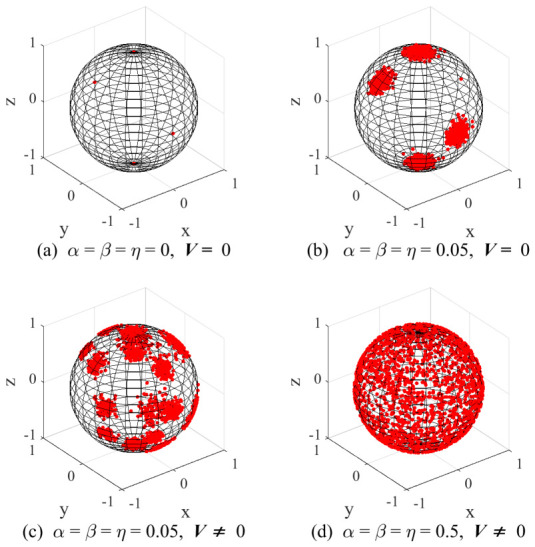
Characteristics of the 4PM constellation.

To verify the security performance of the proposed scheme, simulation and analysis are presented as follows. The simulations are implemented on a PC equipped with an Intel Core i9 processor, 32 GB of RAM, and the Windows 10 operating system. In the example, the length of the simulated signal is 2048 bits. Quadrature phase shift keying (QPSK) is employed as the APM modulation scheme, 4PM is adopted as the polarization modulation mode, and the DL-MPWFRFT transform is applied to the signal. In the simulations, the transform parameter values are given in Table 1, and the variation characteristics of its 2D and 3D constellations are shown in Figure 9 and Figure 10, respectively.

As can be observed from Figure 9, with the variation of DL-MPWFRFT transform parameter values, the constellation diagram of the QPSK signal undergoes rotation and diffusion, and its distribution gradually approaches a Gaussian distribution. This phenomenon significantly increases the difficulty for Eve to demodulate the signal through parameter scanning and achieves more concealed information transmission. Taking Figure 9c as an example, when the QPSK signal is processed by the DL-MPWFRFT with specific parameters, the resulting constellation exhibits a distribution similar to 16QAM. This similarity can cause confusion for Eve, further enhancing the anti-interception performance of the signal.

It can be seen from Figure 10 that the 4PM signal, originally located at four discrete points on the Poincare sphere, is redistributed over the sphere after DL-MPWFRFT processing. Unlike the planar constellation variation of QPSK in Figure 9, this result reflects the concealment effect in the polarization domain, where the clear polarization-state boundaries are weakened and the original 4PM structure becomes difficult to identify. Therefore, DL-MPWFRFT not only disguises conventional amplitude-phase constellations but also obscures polarization-domain signal features, further supporting covert transmission.

### 4.2. Constellation Characteristics of FH-DL-MPWFRFT

By increasing the number of transformation parameters, DL-MPWFRFT enhances the complexity of the transform system, thus lowering the interception probability of the signal for unintended receivers. However, simulation results show that when the transformation order is small, such as 0.05, the signal constellation cannot achieve sufficient rotation and spreading, and there is still a possibility of signal interception by non-intended receivers. Therefore, we further introduce FH phase scrambling into signal modulation, with the fractal coefficient and the initial value set to 4 and 0.9, respectively. To guarantee sufficient chaos of the generated sequence, the number of iterations is set to be greater than 200.

**Figure 11:**
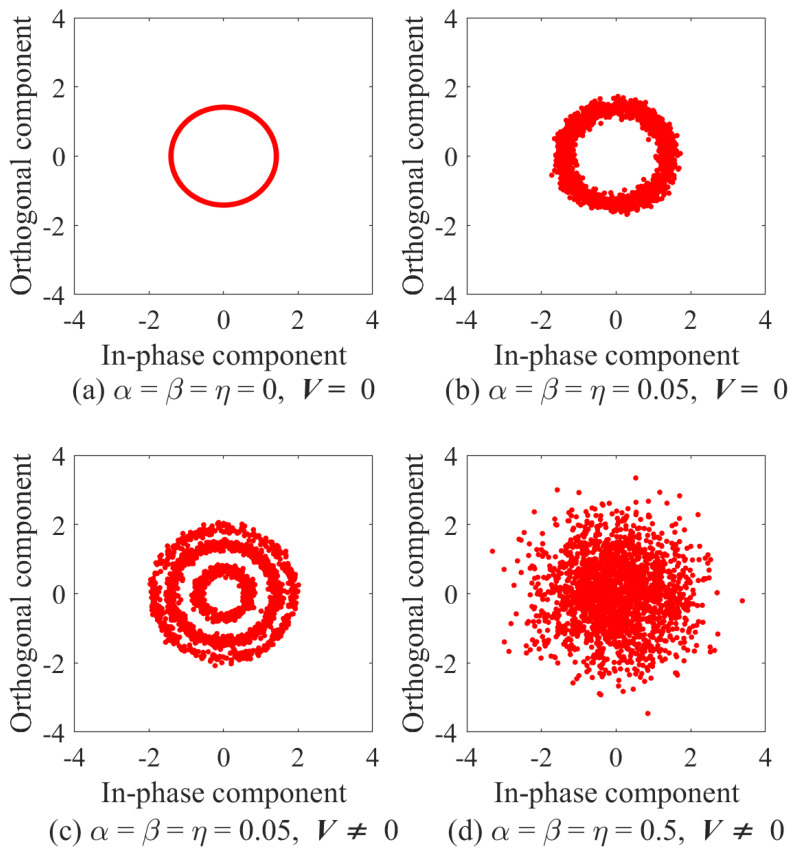
QPSK Constellation processed by FH-DL-MPWFRFT.

**Figure 12:**
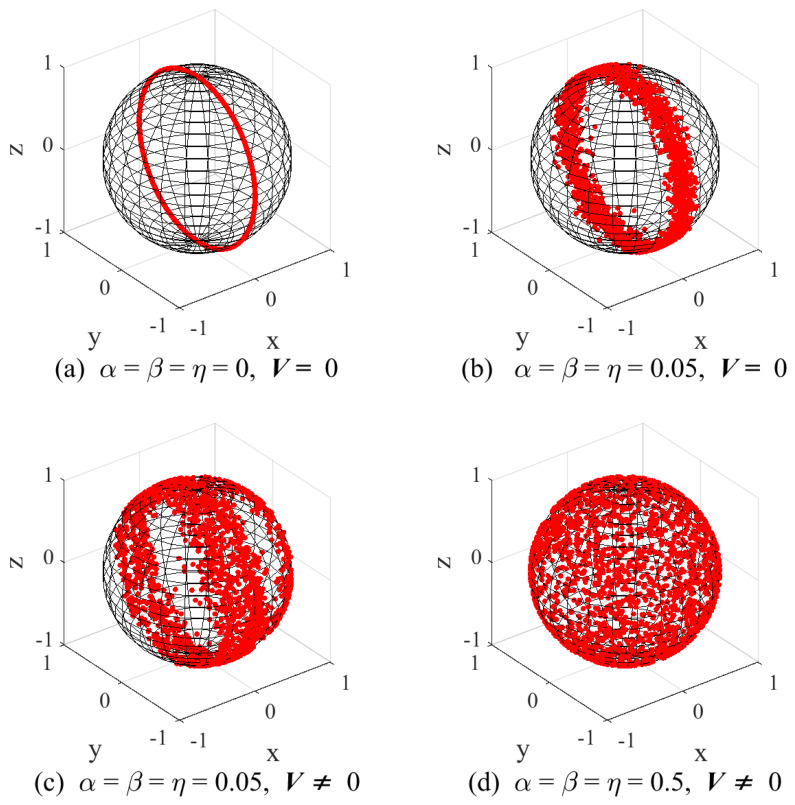
4PM constellation processed by FH-DL-MPWFRFT.

The constellation distribution characteristics of QPSK and 4PM modulated by FH-DL-MPWFRFT are illustrated in Figure 11 and Figure 12, respectively. In Figure 11, the constellation exhibits an approximate Gaussian distribution after processing, with scattered and random constellation points. Currently, modulation recognition based on high-order cumulants (HOCs) is a popular blind detection technique, which is mainly used to identify non-Gaussian signals. However, this method fails to detect Gaussian-like signals effectively, since their HOCs are zero. Therefore, signals processed by FH-DL-MPWFRFT exhibit strong anti-eavesdropping capability.

Similarly, for the QPSK signal shown in Figure 12, it can be observed that after further processing with FH phase scrambling, the 4PM constellation points rotate randomly, which increases the difficulty of signal demodulation for Eve. In addition, taking Figure 12d as an example, the 4PM constellation has been sufficiently disordered after DL-MPWFRFT processing. After further applying FH phase scrambling, there is no obvious change compared with the previous constellation. This makes it difficult for Eve to judge whether the legal receiver has adopted frequency-hopping phase scrambling, leading to errors in the selection of demodulation methods, thereby further improving the secure transmission performance of information.

### 4.3. Anti-Scanning Performance of FH-DL-MPWFRFT

According to the analysis in Section 4.2, Gaussian-like signals cannot be effectively demodulated by HOC-based methods, so Eve can only attempt recovery through parameter scanning. Here, Eve is assumed to know that the legitimate nodes employ the FH-DL-WFRFT scheme. Two scenarios are considered: unknown transform orders and scaling vectors with a known FH phase scrambling factor, and an unknown FH phase scrambling factor with known transform orders and scaling vectors.

#### 4.3.1. Scenario I: Effects of Transform Orders and Scale Vectors

**Figure 13:**
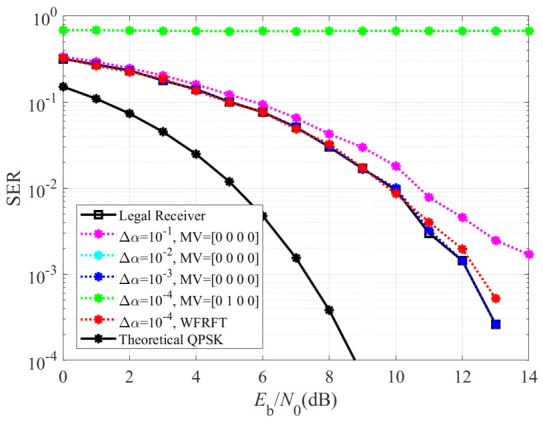
Anti-scanning performance of QPSK signal (role of α and MV).

**Figure 14:**
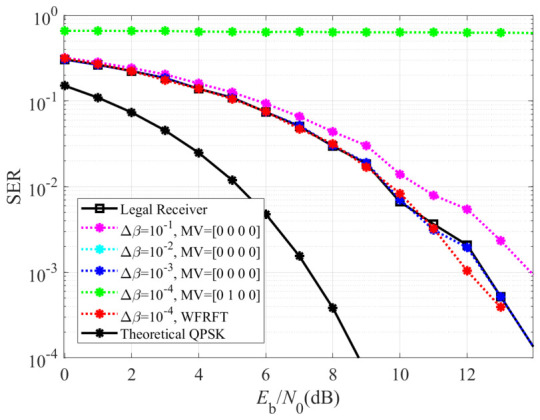
Anti-scanning performance of QPSK signal (Role of β and MV).

**Figure 15:**
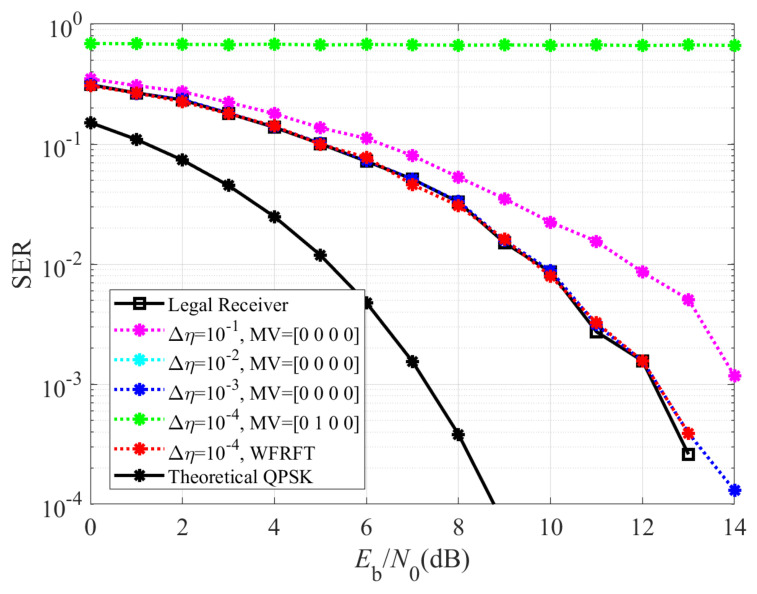
Anti-scanning performance of QPSK signal (Role of η and MV).

**Figure 16:**
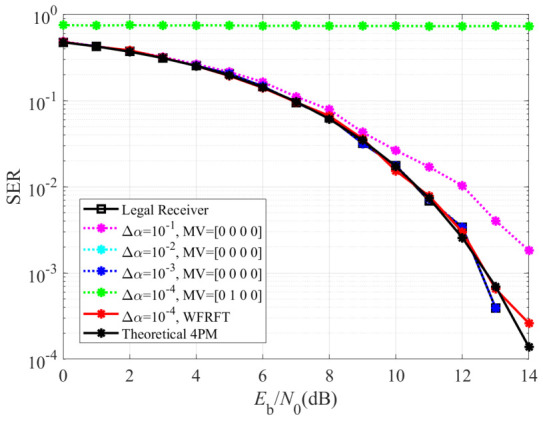
Anti-scanning performance of 4PM signal (Role of α and MV).

**Figure 17:**
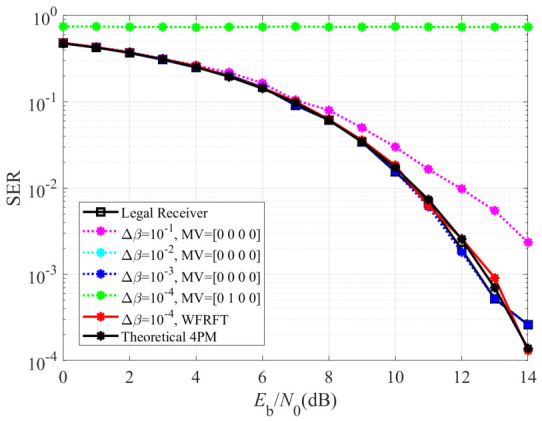
Anti-scanning performance of 4PM signal (Role of β and MV).

**Figure 18:**
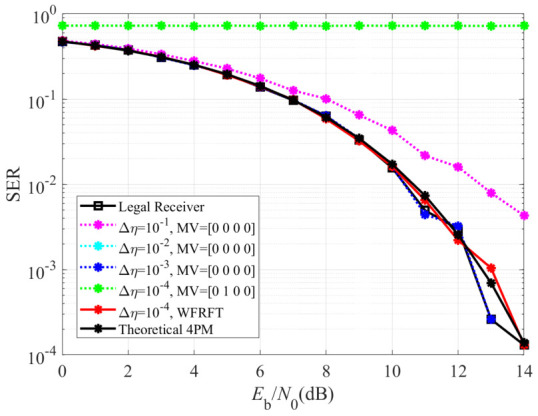
Anti-scanning performance of 4PM signal (Role of η and MV).

From Figure 13, Figure 14, Figure 15, Figure 16, Figure 17 and Figure 18, the receiver symbol error rate (SER) curves derived via the control variable method are presented. In the simulations, α=0.5, $v_{0}=0.9$, and 50 Monte Carlo trials are used to obtain stable performance curves. At Eve’s side, MV and NV are set to $\left[0\;\;0\;\;0\;\;0\right]$ when the transform order error is $10^{-1}$, $10^{-2}$, and $10^{-3}$; when the error is $10^{-4}$, $\mathrm{MV}=\left[0\;\;1\;\;0\;\;0\right]$ and $\mathrm{NV}=\left[0\;\;0\;\;0\;\;0\right]$. In addition, the standard WFRFT method is also included for comparison.

Figure 13, Figure 14 and Figure 15 show the anti-scanning performance of QPSK with respect to transform orders and scaling vectors. As can be observed, the SER curves of the QPSK signal are higher than the theoretical SER. This phenomenon arises because the demodulation of polarization parameters is corrupted by noise, and the resulting SER of the polarization parameters is propagated to the QPSK signal through (5) in the PSM process, thereby yielding an elevated SER relative to the theoretical value. When the transform order discrepancy between Eve and Bob is within 0.01, the SER changes only slightly; once it exceeds this range, Eve’s SER increases rapidly, indicating degraded demodulation and improved anti-interception capability. Furthermore, even under a small transform order error, changing one element of MV will cause a sharp SER increase, showing the strong sensitivity of the proposed scheme to scaling-vector mismatch. Compared with standard WFRFT, FH-DL-MPWFRFT achieves better anti-scanning performance.

Figure 16, Figure 17 and Figure 18 further show that the second-layer parameters also significantly affect 4PM demodulation. For the transform order, the scanning threshold is 0.001, and deviation within ±0.001 keeps SER close to the theoretical value; beyond this threshold, SER rises sharply and may even lead to demodulation failure. For polarized 4PM signals, polarization phase descriptors are directly processed from channel-transmitted signals, ensuring accurate matching with actual signal phase characteristics and avoiding additional SER loss. Thus, legitimate users with correct parameters have SER consistent with the theoretical value. In contrast, Eve cannot concentrate signal energy or recover phase information under parameter mismatch, leading to much higher SER and demonstrating the anti-scanning capability of the proposed scheme.

#### 4.3.2. Scenario II: Effects of FH Phase Scrambling Factor

**Figure 19:**
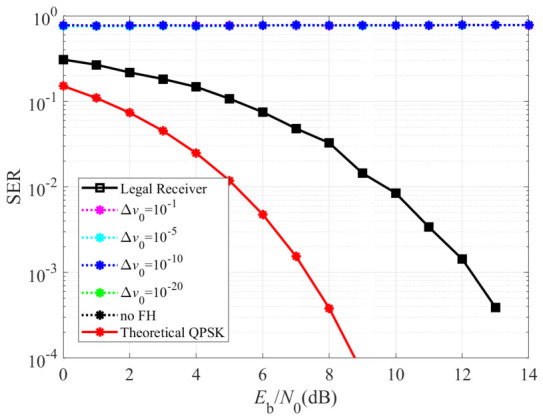
Anti-scanning performance of QPSK signal (Role of the initial value $v_{0}$).

**Figure 20:**
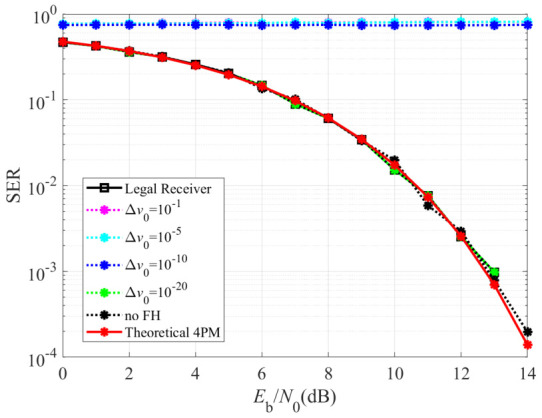
Anti-scanning performance of 4PM signal (Role of the initial value $v_{0}$).

To isolate the effect of the FH phase scrambling factor, Figure 19 and Figure 20 consider a stringent eavesdropping condition in which Eve has already obtained the modulation scheme and the DL-MPWFRFT parameters, including the transform orders and scaling vectors. Under an ideal satellite channel, the remaining uncertainty lies only in the initial value $v_{0}$ of the FH phase scrambling sequence. The no-FH scheme is also included as a baseline to highlight the contribution of this additional scrambling dimension.

For this remaining unknown parameter, $\Delta v_{0}$ in Figure 19 and Figure 20 denotes the scanning error between Eve’s estimated initial value and the true value used by the legitimate receiver. To quantify the difficulty of resolving this remaining uncertainty, the cracking cost under exhaustive scanning is defined as $\varpi_{v}\left(\varepsilon_{v}\right)=1/\varepsilon_{v}$, where $\varepsilon_{v}$ is the scanning resolution over the normalized interval $v_{0}\in \left(0,1\right)$. Successful cracking is considered achieved when Eve’s SER approaches that of the legitimate receiver under the same channel condition. The results show that the SERs of QPSK and 4PM remain around 0.4 to 0.5 for $\Delta v_{0}=10^{-1}$, $10^{-5}$, and $10^{-10}$ and approach the legitimate SER only when $\Delta v_{0}$ reaches the order of $10^{-20}$. This indicates that effective demodulation requires a scanning resolution of approximately $\varepsilon_{v}\leq 10^{-20}$, corresponding to about $10^{20}$ candidate hypotheses for $v_{0}$ alone. Therefore, compared with the no-FH baseline, the FH phase scrambling mechanism introduces an additional high-precision scanning dimension and significantly enhances the waveform-level anti-interception performance.

## 5. Conclusions

In this paper, a covert communication scheme based on FH-DL-MPWFRFT is proposed for satellite–ground-integrated sensor networks. First, on the basis of the definition of MPWFRFT, the DL-MPWFRFT scheme is defined by introducing additional transform layers, which further expands the transform-parameter space and increases the demodulation difficulty for Eve. Then, inspired by the FH phase scrambling mechanism, the physical implementation and corresponding signal characteristics of FH-DL-MPWFRFT are investigated. Furthermore, a polarization-based covert satellite communication system is constructed, and its security performance is verified through numerical simulations under a passive eavesdropping model, where Eve is assumed to know the public modulation format but not the confidential transform and scrambling parameters. The simulation results demonstrate that FH-DL-MPWFRFT achieves superior waveform-level anti-interception performance. By rotating and diffusing the signal constellation, the proposed scheme enhances anti-scanning capability, reduces the eavesdropping probability, and consequently improves the physical-layer secure transmission performance of the system. Since the confidential parameters can be dynamically updated with the FH sequence and transform-layer configuration, estimating the instantaneous parameter set remains challenging for Eve; nevertheless, robustness against adaptive and machine learning-based eavesdroppers will be further investigated in future work.

## Figures and Tables

**Table 1:** Transform parameter values of DL-MPWFRFT in Figure 9 and Figure 10.

Legend	α	*V*	β	$V^{\prime }$	η	$V^{\prime \prime }$
(a)	0	$\begin{array}{c} \left[0\;0\;0\;0\right]\\ \left[0\;0\;0\;0\right] \end{array}$	0	$\begin{array}{c} \left[0\;0\;0\;0\right]\\ \left[0\;0\;0\;0\right] \end{array}$	0	$\begin{array}{c} \left[0\;0\;0\;0\right]\\ \left[0\;0\;0\;0\right] \end{array}$
(b)	0.05	$\begin{array}{c} \left[0\;0\;0\;0\right]\\ \left[0\;0\;0\;0\right] \end{array}$	0.05	$\begin{array}{c} \left[0\;0\;0\;0\right]\\ \left[0\;0\;0\;0\right] \end{array}$	0.05	$\begin{array}{c} \left[0\;0\;0\;0\right]\\ \left[0\;0\;0\;0\right] \end{array}$
(c)	0.05	$\begin{array}{c} \left[0\;3\;0\;0\right]\\ \left[0\;0\;0\;2\right] \end{array}$	0.05	$\begin{array}{c} \left[0\;3\;0\;0\right]\\ \left[0\;0\;0\;2\right] \end{array}$	0.05	$\begin{array}{c} \left[0\;0\;0\;0\right]\\ \left[0\;0\;0\;0\right] \end{array}$
(d)	0.5	$\begin{array}{c} \left[0\;3\;0\;0\right]\\ \left[0\;0\;0\;2\right] \end{array}$	0.5	$\begin{array}{c} \left[0\;3\;0\;0\right]\\ \left[0\;0\;0\;2\right] \end{array}$	0.5	$\begin{array}{c} \left[0\;3\;0\;0\right]\\ \left[0\;0\;0\;2\right] \end{array}$

## Data Availability

The original contributions presented in this study are included in the article. Further inquiries can be directed to the corresponding author.
